# A genome-wide SNP-SNP interaction analysis exploring novel interacting loci associated with the risk of recurrence in colorectal cancer

**DOI:** 10.1371/journal.pone.0321967

**Published:** 2025-06-18

**Authors:** Aaron A. Curtis, Yajun Yu, Megan Carey, Yildiz E. Yilmaz, Sevtap Savas

**Affiliations:** 1 Division of Biomedical Sciences, Faculty of Medicine, Memorial University, St. John’s, NL, Canada; 2 Institute of Cardiovascular Research, Southwest Medical University, Luzhou, Sichuan, China; 3 Department of Mathematics and Statistics, Faculty of Science, Memorial University, St. John’s, NL, Canada; 4 Division of Population Health and Applied Health Sciences, Faculty of Medicine, Memorial University, St. John’s, NL, Canada; 5 Discipline of Oncology, Faculty of Medicine, Memorial University, St. John’s, NL, Canada; Curtin University Bentley Campus: Curtin University, AUSTRALIA

## Abstract

**Background:**

Genetic factors can influence and predict patient outcomes. The association of interactions of germline SNPs with patient outcomes is an understudied area of prognostic research. In this study, we applied the first genome-wide SNP-SNP interaction analysis in relation to colorectal cancer outcomes.

**Objectives:**

Our objective was to explore interacting SNP loci at the genome-wide level that predict the risk of local or distant recurrence (RMFS) in a cohort of stage I-III colorectal cancer patients from the Canadian province of Newfoundland and Labrador.

**Methods:**

The patient cohort consisted of 430 unrelated Caucasian patients. Genetic and medical data was collected previously and the genetic data consisted of a total of 384,415 genotyped SNPs. The PLINK epistasis function was utilized to examine pairwise SNP interactions. Select interactions were assessed by multivariable Cox-regression models, adjusting for established clinical covariates. Genomic regions identified were explored for additional interactions. Published databases were utilized to retrieve biological information about the loci identified.

**Results:**

After Bonferroni correction for multiple testing, no interaction remained significant. We present the top 20 interactions. The interaction p-values ranged from p = 1.37E-8 to p = 2.14E-9 in this set. Interactions were also tested by multivariable Cox regression models including established clinical covariates. Many of the SNPs were intronic and some of them were functional (e.g., expression quantitative expression loci). Analysis of the other SNPs in the same genomic regions as the interacting SNPs led to the identification of three additional interaction models.

**Conclusions:**

We present the results of the first genome-wide SNP-SNP interaction analysis in colorectal cancer outcomes. While no SNP-SNP interaction remained significant after correction for multiple testing, our methodology emphasizes the additional knowledge that can be obtained using interaction analyses while studying prognostic markers.

## Introduction

Colorectal cancer is one the most common cancers globally [1–3]. As in the case of many complex diseases, in colorectal cancer there has been a great interest in identifying prognostic biomarkers (including genetic variants such as Single Nucleotide Polymorphisms [SNPs]) [4]. The past decade has witnessed tremendous progress in our fundamental understanding of biology, health, and disease, as well as in technology, which has allowed researchers to conduct larger and more challenging studies, such as genome-wide association studies (GWASs).

While the GWAS–which often examines individual associations of SNPs with an outcome–is a widely applied method [5–10], it is important to move beyond it. This is because GWAS identified variants usually explain only a small fraction of the phenotypic variation among patients, suggesting that there are genetic mechanisms or variations still waiting to be discovered (i.e., “*missing heritability*” [11–14]). Genetic interactions are one of the possible mechanisms that can help elucidate the missing heritability. In simple terms, interactions (sometimes also referred to as epistasis) are when the effect of a variant on a phenotype is dependent on another variant, where its effect may increase (synergistic interaction) or decrease (antagonistic interaction) in the presence of the other locus [11–17]. As GWASs only examine the effect of one SNP at a time, they cannot identify these interactive effects.

A number of methods have been developed to examine interactions [14,18–29] and further development in this area is expected, including further artificial intelligence-based methods [29]. Since interaction analyses are advanced when compared to one-SNP-at-a-time type analyses, examining interactions can provide unique, novel, and exciting information that can support current studies and further progress prognostic research. Interactions, however, are not well studied in colorectal cancer prognosis. One of the reasons for this is the computational load required to examine interactions and a lack of availability of tools that can handle the large-scale data that is common in genome-wide settings. A few interaction studies in colorectal cancer outcomes, some led by our lab, have been published [30–35]; while these studies opened new ways to examine prognostic biomarkers, they were restricted to candidate genes and/or pathways, missing the opportunity to provide a more comprehensive landscape of the interactions at the genome-wide level.

In the presented study, our aim was to explore genome-wide SNP-SNP interactions that may be associated with the 5-year (local or distant) recurrence risk in colorectal cancer. Our findings underline the potential utility of examining SNP-SNP interactions in biomarker research, can inform future studies, and can inspire other groups to integrate interaction analyses into their studies.

## Methods

### Ethics statement

This study has been approved by the provincial Health Ethics Research Board (HREB #2018.051; #2009.106). As this study was a secondary use of data study, HREB waived the requirement for patient consent. For this study, authors did not require access to information that could identify individual participants during or after data collection. However, previously Megan Carey had access to patient identifiers while updating the outcome data (HREB # 15.006).

### Patient cohort, SNP genotyping and clinical data

The patient clinical and genetic data used in this study were collected by the Newfoundland Familial Colorectal Cancer Registry (NFCCR) investigators between January 1, 1999 and January 24, 2018. The data used in this study was accessed between October 22, 2022 and July 9, 2024. In brief, the NFCCR recruited around 750 patients diagnosed with colorectal cancer between January 1999–December 2003 [36]. Genome-wide SNP genotype data was previously obtained using the Illumina® Omni1-Quad human SNP genotyping platform at a genotyping facility (Centrillion Biosciences, USA) [7]. DNA extracted from non-tumor (e.g., blood) samples was used in genotyping reactions. Patients whose genotype data failed the standard quality control (QC) measures as well as those patients who were non-Caucasian and 1^st^-, 2^nd^-, or 3^rd^-degree relatives were removed from the patient cohort, leaving 505 patients with genome-wide SNP genotype data (consisting of 729,737 SNPs) [7]. Clinical and demographic data was collected using a variety of resources over time, including medical charts, patient questionnaires, and data from local tumor registries [36–38]. The last follow up date was January 24, 2018 [38].

PLINK uses logistic regression in its epistasis analysis. Therefore, we processed the outcome data to fit this method. The clinical endpoint used for this study was 5-year recurrence or metastasis free survival (RMFS) status, where events were defined as recurrence or metastasis at or before 5 years after diagnosis of colorectal cancer. Initially, patients who did not experience recurrence or metastasis and whose last follow-up time was before 5 years were removed from the dataset, as their survival at 5 years was unknown (censored patients; n = 11). Stage IV patients were also removed from the data as our study focuses on the risk of recurrence or metastasis and stage I, II, and III patients are susceptible to these outcomes (i.e., stage IV patients would already have metastases at the time of diagnosis). Patients with missing covariate data were also excluded. After these data processing steps, there remained 430 stage I-III patients in the cohort for the PLINK interaction analysis. Baseline features for this patient cohort are shown in **Table 1**.

**Table 1:** Baseline characteristics of the patient cohort included to the PLINK epistasis analysis.

Variable	N (Total 430)	%
**Location**		
Colon	273	63
Rectum	157	37
**Stage**		
I	84	20
II	188	44
III	158	37
**Baseline Adjuvant Chemotherapy**		
Yes	252	59
No	178	41
**Baseline Adjuvant Radiotherapy**		
Yes	118	27
No	312	73
**Rounded 5-year RMFS Status**		
0 (no recurrence or metastasis)	322	75
1 (recurrence and/ or metastasis)	108	25
RMFS Follow-up Time* (Years)	Median: 15; 95% CI: 14.7–15.3	

N: Number of patients; RMFS: Recurrence-Metastasis-Free Survival. *Calculated by reverse Kaplan-Meier method [40].

### SNP genotype data extraction and quality control measures

All genotype data processing prior to analysis was performed using PLINK 1.9 [39]. Genotypes of SNPs were extracted for the patient cohort using PLINK with the following standard parameters: Missing genotypes = 0, Minor Allele Frequency (MAF) ≥ 0.05, Hardy-Weinberg Equilibrium (HWE) p-value > 0.0001. Genotype data was restricted to autosomal chromosomes, as association analysis of genotypes of the variants on sex chromosomes requires special approaches in cohorts including both men and women, such as ours [41].

The SNP dataset, once extracted, was then subject to pruning. Linkage Disequilibrium (LD)-based pruning was performed using the PLINK commands --indep-pairwise 50 5 0.8 and --extract [42]. This removed one SNP of each pair of SNPs with an r^2^ greater than 0.8, within a window of 50 SNPs, moving the window by 5 SNPs each step. As a result, 384,415 SNPs were included in the final data set.

### PLINK epistasis function

Epistasis analysis was performed using PLINK 1.9. [39] and the --epistasis command. With this command, PLINK performs logistic regression analysis for every pair of SNPs in the dataset. PLINK was chosen for this analysis due to its speed, robustness, memory efficiency, and simplicity. The output of the --epistasis command was retrieved and stored in two files (an epi.cc and an epi.cc.summary file). As a result of this epistasis analysis, 73,887,253,905 interactions were tested.

### Statistical analyses

After Bonferroni correction for multiple testing, none of the interaction p-values remained significant. We report the 20 SNP pairs identified in the epi.cc output file with the lowest interaction p-values (“top 20 SNP pairs”). Genotype data was extracted for these pairs using an additive genetic model with the PLINK command --recodeA.

We constructed univariable Cox regression models for the top SNPs as well as two-SNP Cox regression models (with interaction term) adjusting for clinical covariates of location (rectum vs. colon), stage (II vs I and III vs I), adjuvant chemotherapy (yes vs no), and baseline radiotherapy (yes vs no) [38]. Time to recurrence data was utilized in Cox regression analyses. During these latter analyses, 11 patients who were censored, and hence removed from the PLINK analysis, were added back to the dataset in order to limit bias. Statistical analyses were done using SPSS (IBM; version 29.0.0.0 (241) for Windows). Bonferroni-adjusted significance thresholds are p < 0.00128 and p < 0.0025 for univariable and multivariable Cox regression analyses, respectively.

### Analysis of the interactions in identified genomic regions

As LD-pruning was performed, which removes one SNP of each high-LD pair from the dataset, only a subset of SNPs in our dataset were directly included in our initial PLINK epistasis analysis. In order to address the possibility that interactions involving the pruned SNPs may have stronger associations with RMFS than the interactions identified in our initial analysis, a subsequent analysis was performed. SNPs which were in LD with those identified in the top 20 interactions of our initial epistasis analysis (PLINK r2 inter-chr function) were used to form a new dataset, along with the 39 unique SNPs from the top 20 interactions (one SNP appeared in two interactions). The PLINK epistasis function was again run using this new dataset. There were 30 models found with a p-value ≤ 1.37E-8 (the lowest p-value identified in the initial PLINK interaction analysis). These models included the original 20 interactions, leaving 10 new models for us to consider. In the latter models, each model included one SNP in common with an original interaction set and a new SNP (that is in high-LD with the second SNP from the original interaction). Among those, only three new alternative interactions had a PLINK interaction p-value lower than the original interaction which it corresponded to – these are noted in this manuscript. For each of these alternative interactions, 2-SNP (with interaction term) Cox regression analyses adjusting for covariates were performed as described above.

### Bioinformatics tools and databases

In order to explore the biological knowledge available about the identified loci, we utilized published resources including RegulomeDB [43], GTEx [44], and PUBMED. RegulomeDB ranks variants based on functionality using a scoring system (rank scores vary between 1–7), where with decreasing rank score, the evidence for functionality increases. For example, a ranking score of 1 means the variant is at least an expression quantitative trait/chromatin accessibility quantitative trait locus (eQTL/caQTL) and a transcription factor binding site (https://regulomedb.org/regulome-help/), and hence, is more likely to have functional consequences compared to a variant with a higher ranking score. GTEx, on the other hand, includes expression and eQTL data for normal tissues. Since GTEx does not include data from rectum, here we present the GTEx data extracted for sigmoid and transverse colon only. We note that since none of the interactions remained significant after correction for multiple testing (*see* *Results*), the results of these analyses should rather be taken as explorative in terms of how they relate to colorectal cancer outcomes, but not definitive.

## Results

**Fig 1:**
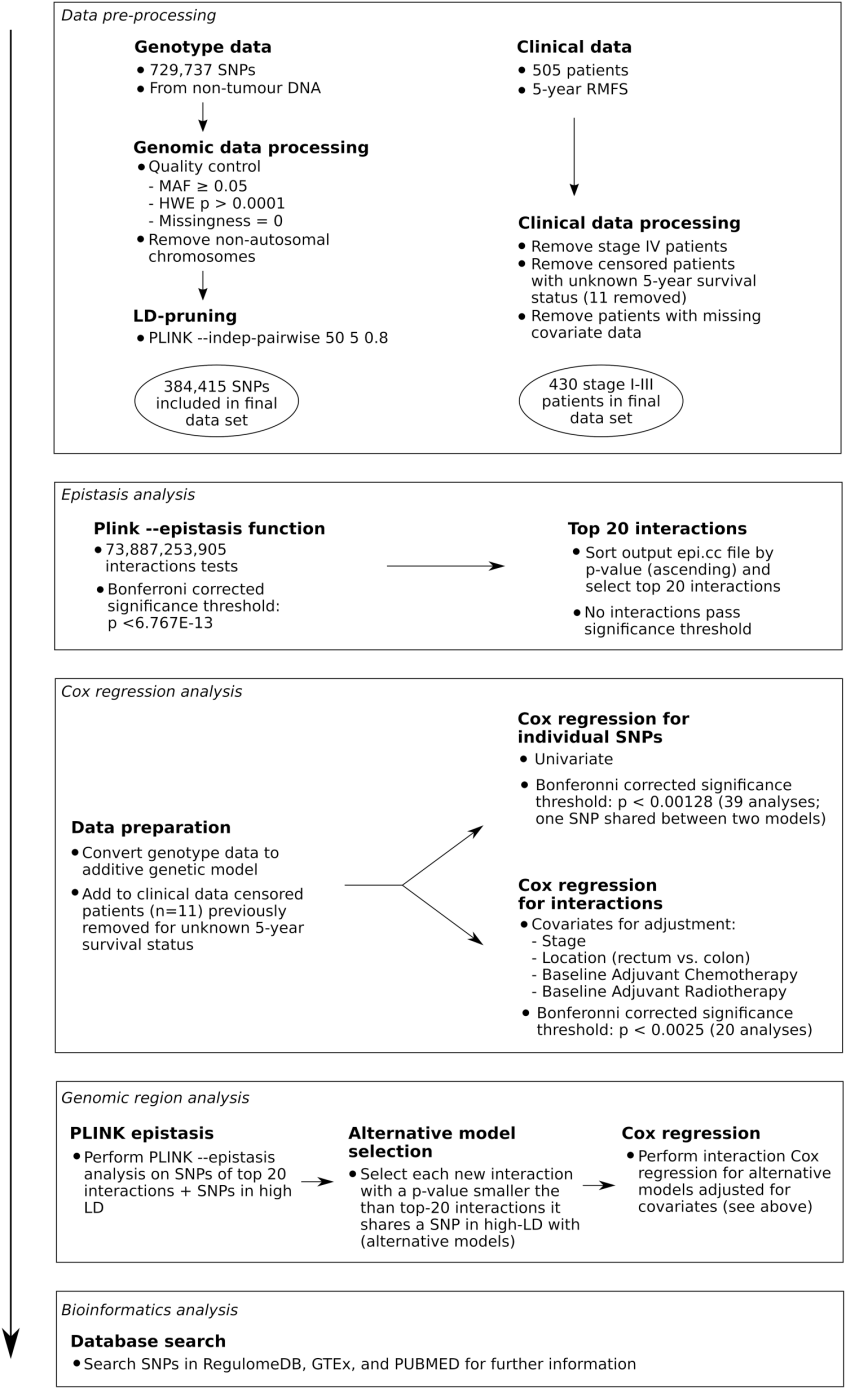
The study workflow. LD: Linkage Disequilibrium; RMFS: Recurrence-Metastasis Free Survival; SNP: Single Nucleotide Polymorphism.

The flow-chart of the study workflow is shown in **Fig 1**. After applying the Bonferroni correction, none of the interactions identified by PLINK remained significant. The top 20 pairs of SNPs with the lowest interaction p-values identified by PLINK are shown in **Table 2**. Except for two cases, interacting SNPs were located in different chromosomes. One SNP (rs742257 on chromosome 1) was identified in two interacting SNP sets.

**Table 2:** PLINK epistasis analysis results for the top 20 interactions (sorted by p-value).

CHR1	SNP1	CHR2	SNP2	OR_INT	STAT	p-value
3	*rs4678497	17	*rs12601535	0.192129	35.8451	2.14E-09
1	*rs742257	11	rs10894641	0.190122	34.9169	3.45E-09
10	*rs11187157	19	*rs12974235	4.97802	34.4279	4.44E-09
2	rs4663576	12	*rs7297676	6.10375	34.3407	4.64E-09
1	***rs2247213	1	rs1266384	4.74791	33.1941	8.37E-09
8	rs7015101	13	rs3850026	0.097299	32.9989	9.25E-09
9	*rs10963949	12	rs9804846	0.175805	32.8939	9.76E-09
2	rs13395344	15	rs11635372	0.244199	32.7967	1.03E-08
2	rs6757680	21	*rs9305669	36.8484	32.7904	1.03E-08
11	***rs35579818	14	rs9671369	0.197519	32.783	1.03E-08
3	*rs2239621	15	rs12901294	7.98319	32.641	1.11E-08
14	**rs12436380	15	rs7181095	4.88222	32.4867	1.20E-08
5	rs12655716	11	kgp5016729	6.07171	32.4841	1.21E-08
2	*rs16850584	7	rs12699307	7.7585	32.4774	1.21E-08
3	rs9855001	9	rs914491	0.203519	32.4508	1.23E-08
6	rs2753172	8	*rs4872541	0.185917	32.4359	1.24E-08
1	*rs742257	11	rs12365003	5.65383	32.3512	1.29E-08
7	*rs10236884	20	***rs6056615	6.88293	32.3418	1.30E-08
1	*rs497915	1	*rs4925659	8.12237	32.3167	1.31E-08
2	rs7575563	13	*rs7998309	8.22868	32.24	1.37E-08

CHR: Chromosome where the SNP is located; SNP: Single Nucleotide Polymorphism; OR_INT: Odds Ratio for the interaction; STAT: Chi-square statistic. SNP rs742257 was identified in two interaction models. *SNPs that have a RegulomeDB score of 1a-1f (S1 Table). **SNP that is an eQTL according to the GTEx database (S1 Table). *** SNP that has a RegulomeDB score of 1a-1f and is also an eQTL according to the GTEx database (S1 Table).

We performed univariable Cox regression (for each SNP; **Table 3**) and multivariable Cox regression analyses (for each SNP pair: **Table 4**) to examine the SNPs’ relationships with the outcome. No SNP was individually associated with RMFS in the univariable Cox regression analysis (Bonferroni adjusted significance threshold < 0.00128). This is expected, as interacting SNPs are not expected to be associated with the outcome individually. In the multivariable models, all interactions had p-values varying between 1.04E-5 and 3.48E-10 (Bonferroni corrected significance threshold is p < 0.0025).

**Table 3:** Univariable Cox regression analysis results for the SNPs in the top 20 list.

SNP	p-value	HR	95% CI for HR
rs497915	0.0932	0.7398	0.5204 - 1.0517
rs742257	0.8921	1.0182	0.7841 - 1.3224
rs2247213	0.4321	0.9031	0.7003 - 1.1646
rs1266384	0.8725	0.9790	0.7557 - 1.2684
rs4925659	0.7286	1.0470	0.8078 - 1.3569
rs7575563	0.0020	0.6023	0.4367 - 0.8307
rs13395344	0.4688	0.9106	0.7066 - 1.1733
rs16850584	0.7794	1.0435	0.7747 - 1.4055
rs6757680	0.1885	0.7751	0.5303 - 1.1330
rs4663576	0.4768	1.0987	0.8478 - 1.4238
rs4678497	0.3100	1.1428	0.8832 - 1.4787
rs2239621	0.5895	0.9275	0.7055 - 1.2192
rs9855001	0.8379	1.0276	0.7916 - 1.3339
rs12655716	0.4489	0.8975	0.6785 - 1.1873
rs2753172	0.8073	1.0359	0.7801 - 1.3757
rs12699307	0.2793	0.8557	0.6453 - 1.1348
rs10236884	0.8238	0.9710	0.7492 - 1.2584
rs7015101	0.8810	1.0239	0.7511 - 1.3960
rs4872541	0.3616	1.1236	0.8748 - 1.4431
rs914491	0.4965	0.9084	0.6888 - 1.1981
rs10963949	0.0741	0.7933	0.6152 - 1.0228
rs11187157	0.1506	0.8295	0.6430 - 1.0703
rs35579818	0.4128	0.8932	0.6817 - 1.1704
kgp5016729	0.5553	0.9226	0.7058 - 1.2059
rs12365003	0.4055	0.8950	0.6891 - 1.1624
rs10894641	0.7766	1.0372	0.8059 - 1.3349
rs9804846	0.1501	1.2323	0.9272 - 1.6378
rs7297676	0.5850	1.0830	0.8135 - 1.4417
rs7998309	0.7625	0.9563	0.7158 - 1.2776
rs3850026	0.3621	0.8857	0.6823 - 1.1498
rs12436380	0.9745	0.9958	0.7675 - 1.2919
rs9671369	0.6859	1.0554	0.8127 - 1.3706
rs11635372	0.3729	1.1244	0.8688 - 1.4550
rs7181095	0.9477	1.0091	0.7693 - 1.3237
rs12601535	0.3118	1.1399	0.8844 - 1.4692
rs12974235	0.6020	0.9346	0.7249 - 1.2051
rs6056615	0.1965	0.8255	0.6170 - 1.1044
rs9305669	0.6541	0.9143	0.6178 - 1.3530
rs12901294	0.6892	0.9416	0.7012 - 1.2645

CI: Confidence Interval; HR: Hazard Ratio; SNP: Single Nucleotide Polymorphism. None of the p-values reached significance when corrected for multiple testing (Bonferroni corrected significance threshold p < 0.00128).

**Table 4:** Multivariable Cox regression analysis results for the SNPs and interactions identified in this study.

Interaction	SNP1	SNP1 p-value	SNP1 HR	SNP1 CI 95% CI for HR	SNP2	SNP2 p-value	SNP2 HR	SNP2 95% CI for HR	Interaction p-value	Interaction HR	Interaction 95% CI for HR
rs12601535*rs4678497	rs4678497	1.1657E-06	2.7822	1.8417 - 4.2029	rs12601535	1.9540E-06	2.5451	1.7322 - 3.7396	9.8468E-08	0.3680	0.2548 - 0.5315
^*μ*^ rs4678497*rs7212295	*rs4678497_C*	*1.2920E-06*	*2.7826*	*1.8387 - 4.2111*	*rs7212295_G*	*1.0191E-06*	*2.6240*	*1.7823 - 3.8634*	*1.0588E-07*	*0.3628*	*0.2496 - 0.5272*
rs10894641*rs742257	rs742257	1.1499E-06	2.7627	1.8344 - 4.1607	rs10894641	9.4060E-05	1.9881	1.4082 - 2.8068	4.4607E-09	0.3310	0.2288 - 0.4790
rs11187157*rs12974235	rs11187157	1.5984E-06	0.3394	0.2183 - 0.5277	rs12974235	1.1795E-04	0.4709	0.3210 - 0.6909	2.2830E-07	2.5821	1.8028 - 3.6984
rs4663576*rs7297676	rs4663576	2.0221E-03	0.5766	0.4065 - 0.8179	rs7297676	9.5192E-05	0.2912	0.1567 - 0.5411	6.7074E-08	3.1026	2.0569 - 4.6799
rs1266384*rs2247213	rs2247213	2.4009E-05	0.3880	0.2501 - 0.6021	rs1266384	3.2757E-04	0.5043	0.3471 - 0.7326	2.2113E-08	2.7931	1.9490 - 4.0028
rs3850026*rs7015101	rs7015101	1.7378E-05	2.7930	1.7481 - 4.4624	rs3850026	1.5856E-02	1.4552	1.0729 - 1.9739	1.4135E-06	0.2624	0.1524 - 0.4520
rs10963949*rs9804846	rs10963949	4.2508E-02	1.4037	1.0115 - 1.9480	rs9804846	1.3059E-05	2.1731	1.5329 - 3.0807	3.3547E-06	0.3609	0.2348 - 0.5546
rs11635372*rs13395344	rs13395344	8.8142E-04	2.0090	1.3318 - 3.0306	rs11635372	1.0965E-04	2.1859	1.4708 - 3.2487	2.9580E-06	0.4273	0.2991 - 0.6103
rs6757680*rs9305669	rs6757680	5.7725E-04	0.4059	0.2429 - 0.6782	rs9305669	2.3546E-03	0.3752	0.1995 - 0.7057	1.4786E-07	10.5782	4.3889 - 25.4962
^***μ***^ kgp11888590*rs6757680	***rs6757680_T***	***3.5088E-04***	***0.3678***	***0.2126 - 0.6365***	***kgp11888590_C***	***3.7784E-03***	***0.4001***	***0.2153 - 0.7437***	***3.1334E-08***	***14.4859***	***5.6203 - 37.3362***
rs35579818*rs9671369	rs35579818	2.2278E-05	2.3481	1.5826 - 3.4838	rs9671369	5.2888E-06	2.3474	1.6258 - 3.3893	3.1171E-09	0.2858	0.1888 - 0.4325
rs12901294*rs2239621	rs2239621	1.5148E-03	0.5320	0.3602 - 0.7857	rs12901294	6.7235E-04	0.4707	0.3049 - 0.7267	1.0386E-05	2.7371	1.7494 - 4.2825
rs12436380*rs7181095	rs12436380	2.5820E-05	0.4194	0.2798 - 0.6286	rs7181095	3.5719E-06	0.3227	0.2000 - 0.5206	2.1529E-09	3.3887	2.2724 - 5.0534
kgp5016729*rs12655716	rs12655716	2.1555E-05	0.3776	0.2409 - 0.5918	kgp5016729	2.8294E-05	0.4339	0.2935 - 0.6414	8.6117E-09	3.3175	2.2053 - 4.9906
rs12699307*rs16850584	rs16850584	6.6366E-04	0.4492	0.2833 - 0.7121	rs12699307	1.2392E-04	0.4296	0.2791 - 0.6614	3.1648E-07	4.1771	2.4149 - 7.2253
rs914491*rs9855001	rs9855001	5.2221E-05	2.2174	1.5076 - 3.2613	rs914491	3.3890E-05	2.5888	1.6512 - 4.0588	9.4235E-08	0.3335	0.2228 - 0.4991
^***μ***^ rs4485715*rs914491	***rs4485715_A***	***8.2027E-05***	***2.1719***	***1.4764 - 3.1950***	***rs914491_G***	***3.4072E-05***	***2.5301***	***1.6311 - 3.9245***	***6.9756E-08***	***0.3302***	***0.2207 - 0.4939***
rs2753172*rs4872541	rs2753172	7.4902E-06	2.4653	1.6611 - 3.6587	rs4872541	6.8317E-05	1.8842	1.3795 - 2.5737	2.3741E-07	0.3213	0.2089 - 0.4943
rs12365003*rs742257	rs742257	1.0379E-05	0.3834	0.2503 - 0.5871	rs12365003	8.4464E-06	0.4011	0.2684 - 0.5996	3.4846E-10	3.6358	2.4295 - 5.4411
rs10236884*rs6056615	rs10236884	6.5256E-05	0.4585	0.3127 - 0.6724	rs6056615	7.2669E-07	0.2873	0.1754 - 0.4706	8.1900E-09	4.1029	2.5389 - 6.6305
rs4925659*rs497915	rs497915	6.0636E-06	0.2206	0.1146 - 0.4245	rs4925659	1.9607E-02	0.6742	0.4842 - 0.9388	2.2989E-07	3.3005	2.0995 - 5.1885
rs7575563*rs7998309	rs7575563	5.4081E-08	0.2704	0.1687 - 0.4332	rs7998309	6.0391E-05	0.3881	0.2444 - 0.6163	6.2034E-08	4.9145	2.7612 - 8.7471

CI: Confidence Interval; HR: Hazard Ratio; SNP: Single Nucleotide Polymorphism; *interaction. ^μ^alternative interacting SNP set identified through analysis of the genomic regions where the original top 20 interacting SNPs were located. Alternative interaction models with lower p-values than their corresponding original interaction are shown in bold. Bonferroni corrected significance threshold is p < 0.0025.

We next examined whether there were interactions with smaller p-values that may have been missed because of SNP pruning, within the genomic regions captured by the identified SNPs. As a result, three new interacting SNP pairs were found (also called “alternative” sets or models in this manuscript). In these new models, one of the SNPs was the same variant between the original and the alternative models): rs4678497 and rs7212295, as an alternative to rs4678497-rs12601535; rs6757680-kgp11888590 as an alternative to rs6757680-rs9305669; and rs4485715 and rs914491 as an alternative to rs9855001-rs914491. In multivariable Cox regression analyses, two of these interactions had a p-value lower than the original interaction (**Table 4**).

S1 Table summarizes the MAFs, genetic locations, associated genes, RegulomeDB rankings, and eQTL information for the SNPs. Many of the SNPs were intronic SNPs and two SNPs were located in coding regions (rs12601535, a synonymous substitution located in *MYO18A*, and rs9671369, a missense variant in the *SYNE3* gene). Twenty SNPs identified in this study were highly likely to be functional (based on RegulomeDB [43] rank of 1a-1f; S1 Table). Interestingly, in some cases both of the interacting SNPs were likely functional (**Table 2**).

Based on the GTEx [44] data, four of the SNPs in the list were eQTLs in sigmoid and/or transverse colon tissues (eQTLs are variants that are associated with the expression levels of certain genes [43]) (rs2247213; rs6056615; rs12436380; and rs35579818 being eQTLs for *LINC01352, HLX-AS1,* and *RP11-295M18.6*; *RSPO4*; *TRIM9*; and *TMEM80*, *HRAS*, and *RNH1* genes, respectively). Additionally, rs9984518 identified in one of the alternative SNP sets was found to be an eQTL for *WRB* in transverse colon. No entry was returned when PUBMED and dbCPCO [45] databases were searched with the IDs of the SNPs identified in this study. Lastly, a literature search showed that many of the genes identified in this study were linked to colorectal cancer development, progression, or outcomes previously (see Discussion).

## Discussion

Colorectal cancer is a common disease worldwide [1–3] and in Canada [46]. New prognostic markers can help prognosis, improve understanding of the biological reasons for variable prognosis among patients, and aid in the development of better therapeutic agents and prognostic tools. In this study, by following a robust and easy-to-apply approach, we aimed to explore the SNP-SNP interactions that are associated with the local or distant recurrence-free survival times in a cohort of colorectal cancer patients from Canada.

Prognostic GWASs are widely performed studies. While they are quite popular, their analytical abilities are restricted to analysis of the relationship of individual variants to an outcome measure. This may potentially lead to missing at least a part of the “heritability” component of prognostic variability, which can be explained by interacting loci [11–14]. Previously, our group [30,31] and others [33–35] have seen this possibility and applied interaction analyses at candidate gene or pathway settings in colorectal cancer. A genome-wide interaction analysis, as we have done here, however, has not been done in colorectal cancer before. Our analysis explored candidate SNP-SNP interactions in relation to recurrence in colorectal cancer. While further studies are needed to validate these findings and the accuracy of the models, and assess the potential short-term and long-term effects of the SNP interactions, we invite all researchers with genetic and RMFS data to test our findings in their datasets.

There may be also a timely opportunity to investigate or confirm the biological features of the SNPs reported in this manuscript, and their relation to disease recurrence in colorectal cancer using experimental approaches. Note that many of the SNPs highlighted in our study were estimated to affect biological functions/gene regulation. In some cases, both SNPs in the interaction set were predicted to be functional (e.g., based on the RegulomeDB scores or GTEx eQTL information). These SNPs are likely “lead SNPs” [47] that can be prioritized for further studies. In addition, literature search showed that some of the genes that SNPs identified here-in were located in (e.g., *LAMB3* [48,49]; *SYNE3* [50]; *HRAS* [51,52]; *DIAPH3* [53]; *NLRP3* [54,55]); *PEBP4* [56]; *GABRG3* [57]; *LGALS8* [58]; *OPCML* [59]; *MYO18A* [60]; *HAUS6* [61,62]; *HLX* [63,64] and some of the genes that were linked to eQTLs identified here-in (e.g., *HRAS* [51,52]) were previously linked to colorectal cancer. Altogether, this information strengthens the potential biological connections of some of the SNPs identified in this study with colorectal cancer.

The key strengths and limitations of this study can be summarized as follows: PLINK [39] is a fast and robust tool and it handled our large dataset reasonably quickly (less than a day), however, the PLINK epistasis function currently cannot adjust for covariates and can examine two SNP interactions only. Therefore, PLINK misses higher-order interactions (3-SNP and higher) and requires follow-up analyses with multivariable modeling to examine whether interactions are independent of established clinical covariates. There is also a need for robust tools that can handle time-to-event analyses while examining large number of interactions. The cohort for this study has a long follow-up time, but it included only Caucasian patients, and as such, results may not be applicable to other ethnicities. X-linked and Y-linked SNPs were excluded from the analysis, therefore additional information can be gained by examining the variants on these chromosomes in future studies. The SNP dataset included common SNPs with a MAF of at least 5%, hence, interactions involving rare variants also remain to be examined. Our cohort size was small and none of the p-values provided by PLINK remained significant after the Bonferroni correction. Therefore, further research in bigger cohorts with comparable characteristics (e.g., ancestry, treatment features) is required to test the results of this hypothesis generating study and before any of these interactions can be included in a prognostic model in the clinic. Lastly, this is the first time such an extensive interaction analysis has been performed in colorectal cancer, and as such, our results encourage new ways to examine prognostic biomarkers beyond the traditional GWAS approach.

## Conclusions

Interaction analyses can identify collective relations of multi-variables in relation to a phenotype. While conducting genome-wide interaction studies is challenging, a few computational methods have been developed that can be utilized for such purposes. Here we present such a study performed using PLINK, the first genome-wide SNP-SNP interaction analysis in colorectal cancer outcomes. Our study brings new depth to the prognostic research, can inform future studies, and is expected to inspire other groups to integrate interaction analyses in their prognostic studies. Further studies building on our findings can also advance prognostic research, identify new interactions, and help address the missing heritability in colorectal cancer prognosis.

## Supporting information

S1 Table Information about the SNPs identified in this study. Chr: Chromosome; eQTL: expression Quantitative Trait Locus; MAF: Minor Allele Frequency; SNP: Single Nucleotide Polymorphism. * This SNP shares the same location as kgp5016729. ** This SNP shares the same location as kgp11888590. ^μ^Alternative interacting SNP identified through analysis of the genomic regions where the original top 20 interacting SNPs were located. ^£^Reference genome: hg19.
(PDF)
