# Supplementary material for: A genome-wide SNP-SNP interaction analysis exploring novel interacting loci associated with the risk of recurrence in colorectal cancer
Source: PLoS One. 2025 Jun 18;20(6):e0321967. doi: 10.1371/journal.pone.0321967 (PMC12176161; doi:10.1371/journal.pone.0321967)
Supplement: S1 Table — Chr: Chromosome; eQTL: expression Quantitative Trait Locus; MAF: Minor Allele Frequency; SNP: Single Nucleotide Polymorphism. * This SNP shares the same location as kgp5016729. ** This SNP shares the same location as kgp11888590. μAlternative interacting SNP identified through analysis of the genomic regions where the original top 20 interacting SNPs were located. £Reference genome: hg19. (PDF) [file pone.0321967.s001.pdf]

Supporting Information

A genome-wide SNP-SNP interaction analysis exploring novel interacting loci associated with the risk of recurrence in colorectal cancer

[1]Aaron. A. Curtis, [2]Yajun Yu, [1]Megan Carey, [3]Yildiz E. Yilmaz, [1,4,5]Sevtap Savas*

[1]Division of Biomedical Sciences, Faculty of Medicine, Memorial University, St. John's, NL, Canada.

[2]Institute of Cardiovascular Research, Southwest Medical University, Luzhou, Sichuan, China.

[3]Department of Mathematics and Statistics, Faculty of Science, Memorial University, St. John's, NL, Canada.

[4]Division of Community Health and Humanities, Faculty of Medicine, Memorial University, St. John's, NL, Canada.

[5]Discipline of Oncology, Faculty of Medicine, Memorial University, St. John's, NL, Canada.

Supporting Table S1. Information about the SNPs identified in this study.

SNP	Chr	[£]Location	MAF	Molecular consequence (dbSNP)	Minor allele/ Major allele	Gene (dbSNP)	RegulomeDB rank	GTEx eQTL in sigmoid colon – target gene	GTEx eQTL in transverse colon – target gene
rs4678497	3	33213119	0.3512	Intron variant	C/T	SUSD5	1f	No	No
rs742257	1	209795302	0.3314	Intron variant	T/C	LAMB3	1f	No	No
rs11187157	10	94502244	0.4163	None	C/T	None	1f	No	No
rs4663576	2	236270060	0.493	None	A/G	None	5	No	No
rs2247213	1	221055463	0.3442	Intron variant (HLX), upstream variant (HLX-AS1)	A/G	HLX, HLX-AS1	1f	Yes - LINC01352; RP11-295M18.6; HLX-AS1	Yes - RP11-295M18.6; LINC01352
rs7015101	8	9718278	0.1988	None	G/T	None	7	No	No
rs10963949	9	19084444	0.4198	Intron variant	T/G	HAUS6	1f	No	No
rs13395344	2	88795168	0.4291	None	A/G	None	7	No	No
rs6757680	2	172815510	0.1547	Intron variant	T/G	HAT1	7	No	No
rs35579818	11	545087	0.3872	Intron variant	C/T	LRRC56	1f	Yes - TMEM80; HRAS	Yes - RNH1; TMEM80; HRAS
rs2239621	3	38173733	0.2814	Intron variant	T/C	ACAA1	1f	No	No

rs12436380	14	51569780	0.4384	None	A/C	None	6	No	Yes - TRIM9
rs12655716	5	92003403	0.2988	Intron variant	T/C	LOC105379082	7	No	No
rs16850584	2	166263511	0.2279	None	C/A	None	1f	No	No
rs9855001	3	167243869	0.486	Intron variant	C/A	WDR49	7	No	No
rs2753172	6	124643000	0.264	Intron variant	T/C	NKAIN2	5	No	No
rs10236884	7	148659616	0.357	2KB upstream variant	A/G	RNY4	1f	No	No
rs497915	1	182406654	0.2058	None	A/G	None	1f	No	No
rs7575563	2	36104933	0.293	None	G/A	None	6	No	No
rs12601535	17	27441077	0.4733	Synonymous variant	G/C	MYO18A	1f	No	No
rs10894641	11	132924682	0.5	Intron variant	C/T	OPCML	7	No	No
rs12974235	19	56097419	0.4488	None	C/T	None	1a	No	No
rs7297676	12	125410562	0.2721	None	T/G	None	1f	No	No
rs1266384	1	236684057	0.3779	Intron variant	G/A	LGALS8	2b	No	No
rs3850026	13	66508344	0.393	None	C/T	None	7	No	No
rs9804846	12	12116926	0.2767	None	A/G	None	4	No	No
rs11635372	15	50137718	0.4663	None	A/G	None	6	No	No
rs9305669	21	40547329	0.1198	3 Prime UTR variant	T/C	PSMG1	1f	No	No

rs9671369	14	95906321	0.3686	Missense variant	A/G	SYNE3	2b	No	No
rs12901294	15	27471426	0.2558	Intron variant	T/A	GABRG3	5	No	No
rs7181095	15	93574819	0.3802	None	T/C	None	5	No	No
kgp5016729	11	123359638	0.3407	NA	G/A	NA	NA	No	No
*rs2846317	11	123359638	NA	Intron variant	NA	GRAMD1B	2b	No	No
rs12699307	7	12130843	0.2779	Intron variant	G/A	LOC124901589	2b	No	No
rs914491	9	2419927	0.3547	Intron variant	G/A	LOC101930053, LOC105375956	7	No	No
rs4872541	8	22602046	0.4256	Intron variant	C/A	PEBP4, LOC124901908	1d	No	No
rs12365003	11	132924414	0.3581	Intron variant	T/C	OPCML	7	No	No
rs6056615	20	995281	0.3012	None	C/A	None	1f	Yes - RSPO4	Yes - RSPO4
rs4925659	1	247603463	0.3965	Intron variant	A/G	NLRP3, LOC124904575	1f	No	No
rs7998309	13	60324353	0.2628	Intron variant	T/C	DIAPH3	1f	No	No
[µ]rs4485715	3	167235455	0.4814	Intron variant	A/C	WDR49	1f	No	No
[µ]rs7212295	17	27431425	0.4663	Intron variant	G/A	MYO18A	6	No	No
[µ]kgp11888590	21	40583166	0.1163	NA	C/T	NA	NA	No	No
**rs9984518	21	40583166	NA	Intron variant	NA	BRWD1	1f	No	Yes - WRB

Chr: Chromosome; eQTL: expression Quantitative Trait Locus; MAF: Minor Allele Frequency; SNP: Single Nucleotide Polymorphism. * This SNP shares the same location as kgp5016729. ** This SNP shares the same location as kgp11888590.

[µ]Alternative interacting SNP identified through analysis of the genomic regions where the original top 20 interacting SNPs were located. [£]Reference genome: hg19.

References

Boyle AP, Hong EL, Hariharan M, Cheng Y, Schaub MA, Kasowski M, *et al*. Annotation of functional variation in personal genomes using RegulomeDB. Genome Res. 2012;22(9):1790–7.

Lonsdale J, Thomas J, Salvatore M, Phillips R, Lo E, Shad S, *et al*. The Genotype-Tissue Expression (GTEx) project. Nat Genet. 2013;45(6):580–5.
